# The Moderating Role of Caregiving on Fear of COVID-19 and Post-Traumatic Stress Symptoms

**DOI:** 10.3390/ijerph18116125

**Published:** 2021-06-06

**Authors:** José Luis Carballo, Ainhoa Coloma-Carmona, Sara Arteseros-Bañón, Virtudes Pérez-Jover

**Affiliations:** Center for Applied Psychology, Miguel Hernández University, Avenida Universidad s/n, 03202 Elche, Spain; sarteseros@umh.es (S.A.-B.); v.perez@umh.es (V.P.-J.)

**Keywords:** COVID-19 fear, post-traumatic symptoms, caregiver, informal caregiver, pandemics

## Abstract

Caregiving has been associated with increased levels of fear and post-traumatic stress symptoms (PTSS) during COVID-19 pandemic. However, there is a lack of studies that analyze when the relationship between fear and PTSS occur, using informal caregiving as a moderator variable. To explore this moderating role, we conducted a cross-sectional online study between November 2020 and January 2021. A total of 503 men and women from the Spanish general population completed the survey. Sociodemographic and Covid-19-related data, fear of COVID-19, PTSS symptoms, and current psychological history were assessed. Prevalence of informal caregiving in the sample was 16.5%. Increased levels of fear and PTSS were found in caregivers compared to non-caregivers. Female gender and high number of COVID-19 related risk factors was also associated with fear and PTSS severity. The moderation analyses showed an interaction effect between caregiving and fear of COVID-19 when predicting PTSS symptoms. Particularly, results showed that informal caregivers reported greater PTSS symptoms, when compared to non-caregivers with same levels of fear of COVID-19. This evidence suggests that being a caregiver could increase the fear’s impact on PTSS severity in the context of pandemics. Further studies with larger samples are needed to confirm these findings.

## 1. Introduction

Several studies have analyzed the COVID-19 pandemic’s effects on mental health of general and vulnerable populations—such as healthcare workers, the elderly, or chronic-disease patients [1,2,3,4,5]. All reviews agree that the pandemic has increased mental health problems globally, with generalized fear as the central feature of its psychological impact [6,7].

Fear is a common emotional response during pandemics [7,8,9,10] and it can have a positive influence on infection control measures and prevention behaviors, such as isolation compliance, mask usage, hand-washing, or social distancing [11]. For this reason, some prevention strategies have intended propagated fear in order to curb the spread of coronavirus and reduce the consequences of the pandemic [12]. However, the use of this strategy, along with strict lockdown measures, has been associated with excessive fear and a lack of sense of security, specially promoted by the media [12,13,14]. As a result of this situation, previous authors found that fear of the COVID-19 and its effects is currently far greater than the negative experiences lived during this pandemic [12]. These findings merit special attention, since besides its adaptive function, excessive and prolonged fear has been associated with greater risk of developing of psychopathology [15,16,17,18].

In this regard, failure to reduce fear responses is one of the major reasons behind the development of post-traumatic stress symptoms (PTSS) [19,20], and increased prevalence of these symptoms have been also found during COVID-19 pandemic [21].

Prevalence of PTSS during COVID-19 and other pandemics—such as Ebola, Zika, or SARS—has been estimated around 20% [22,23]. The heightened prevalence of stress-related disorders after COVID-19 outbreak has been linked with higher fear of becoming infected, along with other variables such as younger age, female gender, or having infected relatives/friends [24,25,26,27,28]. Moreover, excessive fear has been also associated with social problems like panic buying, stigmatization of healthcare workers, and xenophobia [29,30,31].

Regarding this COVID-19-related stress reactions, different authors have emphasized that fear experienced during COVID-19 pandemic not only represents being scared of getting infected. In fact, a recent study has found that fear as regards the health consequences of COVID-19 only constitute a third of the anxieties reported by the European population [12]. In this sense, fear appears to be a multidimensional phenomenon which involves different domains—including bodily, interpersonal, behavioral, and cognitive features [32,33]. Given its importance in the context of a pandemic, several studies have examined which variables increase the risk of experiencing intense fear. Again, female gender as well as lower educational level, intolerance of uncertainty or perceived vulnerability to the disease have been identified as strong risk factors for fear, not only in current COVID-19 pandemic, but also in the previous aforementioned epidemics [8,17,30,34,35,36]. Fear is also associated with lower self-efficacy and insecure attachment, especially in the use of avoidance behaviors to manage anxiety and as preventive strategies [37,38]. Besides these factors, previous evidence also suggests a relationship between high levels of fear, the perceived risk for loved ones, and concerns about infecting family members, especially in those who have a caregiving role [17,39,40,41].

Informal caregiving, which is defined as the unpaid care provided to dependent relatives or family members [42], seems to increase even more the psychological consequences of the COVID-19 pandemic. Greater levels of anxiety, depression and even somatic symptoms have been found in this population [40,43,44,45,46], while its caregiving intensity and burden increased during this period [47,48]. Due to the complete closure of day-care centers, the loss of support services and the saturation of healthcare capacities, informal caregivers had played an even more important role during COVID-19 pandemic [43,49,50]. For this reason, informal care has been considered an additional stressor that can negatively affect the physical and mental health of an already vulnerable population [43,51,52,53].

Although being considered a stressor, there is still weak evidence about the specific role of this variable in the development of stress-related disorders during COVID-19 pandemic. More particularly, to the best of our knowledge, there are no studies that examine in which way informal caregiving impacts the relationship between fear of COVID-19 and post-traumatic symptoms. For this reason, the aim of this study was to examine the potential moderator effect of caregiving in the relationship between both variables.

Based on the aforementioned evidence, the following exploratory hypotheses were tested:

**Hypothesis** **1.** *Higher levels of fear of COVID-19 and greater PTSS symptoms will be significantly associated with female gender, younger age, lower education levels, informal caregiving, and higher number of COVID-19-related variables.*

**Hypothesis** **2.** *High levels of fear of COVID-19 will be significantly associated with greater PTSS severity.*

**Hypothesis** **3.** *Informal caregiving would moderate the association between fear of COVID-19 and PTSS severity.*

## 2. Materials and Methods

### 2.1. Study Design and Participants

This cross-sectional study was approved by the Committee of Research and Ethics of the Miguel Hernández University of Elche (reference number: DPS.JCC.01.20).

Sampsize program [54] was used to calculate the minimum sample size. In Spain, prevalence of informal caregiving is estimated at 16% [55]. Based on this rate, the minimum sample size required for this study was 207 (with a 5% margin of error and 95% confidence level). Inclusion criteria were as follows: (1) age ≥ 18, (2) living in Spain during COVID-19 crisis, and (3) signing informed consent before participating in the study.

Potential participants were recruited between November 2020 to January 2021 using a multi-modal strategy. First, a survey was distributed via social media platforms. To minimize the bias of nonprobability sampling, 10 initial participants (‘seeds’) were selected to initiate the survey link distribution. Seeds were selected based on gender, age category, geographical location, and occupational status. Then, survey was distributed via the mailing lists of the Miguel Hernández University. Participants were invited to participate in the study using the following statement: “Researchers of the Miguel Hernández University want to know how the COVID-19 pandemic is affecting you. For this reason we are developing a tool for preventing emotional problems during the pandemic. Tracking your mood during these days can help us improve the accuracy of the tool that will be used by healthcare professionals. Would you like to collaborate in our study? Please followed this link to our website for more details”. All participants were directed to an external survey website (preventept.com (accessed on 1 January 2021)), which host information about the aim of the study, the Participant Information Statement text and a link to the online questionnaire. Chatbot technology of the SurveySparrow platform was used to display survey questions in a conversational manner, which has been pointed out as a cost-effective assessment method [56,57]. These recruitment strategies yielded a total sample of 503 individuals from the Spanish general population.

### 2.2. Measures

Participants provided information about age, gender, educational level, and occupational risk of exposure to COVID-19 (e.g., frontline responders or healthcare workers).

Informal caregiving was ascertained by asking participants: “Do you assisted a family member or relative who has health problems without receiving any salary?”. Response alternatives were: (1) yes; (2) not now, but I have assisted a family member/relative during the last 12 months; and (3) no. Only participants who answer ‘no’ to this question were classified as non-informal caregivers.

Post-traumatic stress symptoms (PTSS) were assessed with the Post-traumatic Stress Disorder (PTSD) Checklist for DSM-5 (PCL-5) [58]. PCL-5 is made up of 20 Likert-type items that assess PTSD symptoms according to DSM-5 diagnostic criteria [59]. Participants rate how much each symptom has bothered them on a five-point scale (0 = not at all, 4 = extremely). As in previous studies [60], items asking about symptoms of reexperiencing and trouble remembering parts of the stressful experience were deleted from the PCL-5, since the pandemic is an ongoing stressor [61,62]. This instrument has been widely used for assessing PTSD symptoms prior and during COVID-19 pandemic [22,63], because of its good psychometric properties. Total score of the scale, ranging from 0 to 72, indicated severity of PTSS symptoms.

Fear of COVID-19 was evaluated using the Fear of COVID-19 Scale (FCV-19S) [7], in its Spanish version [64]. This unidimensional measure includes seven items with Likert-type response options ranging from 1 (strongly disagree) to 5 (strongly agree). Scores in each item of the scale item are adding up to a total score of 7 to 35 points, with higher values indicating greater fear of COVID-19. The Spanish version of the FCV-19S has shown acceptable internal consistency and test-retest reliability (α = 0.82 and ICC = 0.72) [64].

Similar to previous research [65], a COVID-19 risk factors index was created by adding up each positive answer (yes) of four ad-hoc items assessing: (1) occupational risk of exposure to COVID-19 (e.g., frontline responders or healthcare workers); (2) self-reported COVID-19 symptoms/diagnosis or hospitalization due to COVID-19; (3) family or relatives who were infected, hospitalized or dead because of COVID-19; and (4) having received psychological treatment during the epidemic. Higher scores indicate the presence of more COVID-19-related risk factors (ranging from 0 to 4).

### 2.3. Analysis Strategy

Data were analyzed using the SPSS 27.0 software. First, means, standard deviations, and bivariate correlations were computed for all variables.

**Figure 1:**
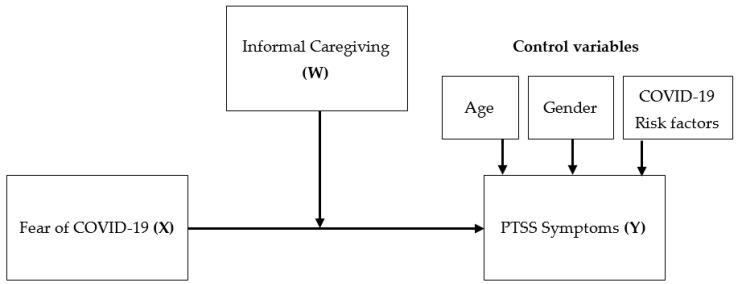
Conceptual diagram of the moderation model.

To test whether caregiving moderates the relationship between fear of COVID-19 (predictor variable) and PTSS symptoms (outcome variable), a simple moderation analysis was also conducted using PROCESS Macro Model 1 [66]. The conceptual model is shown in Figure 1. Variables that were significantly correlated with PTSS symptomatology, were included as covariates in moderation analysis. Post-hoc simple slope analyses were performed to estimate conditional effects of the moderator variable. The interaction effect (Fear of COVID-19 × Caregiving) was considered significant when 95% confidence intervals (CIs) did not include zero [66]. Bootstrapping resampling technique (with 10,000 replications) was used to estimate 95% CIs and continuous variables were mean centered to avoid potential multicollinearity effects [67]. The confidence level was set at 95%.

## 3. Results

### 3.1. Sample Characteristics

Table 1 shows the demographic, psychological and COVID-19 related characteristics of the total sample (N = 503). Participants’ mean age was 35.54 ± 12.79 (ranging 18–75 years) and 82.50% (*n* = 415) were female. Regarding informal caregiving during COVID-19 pandemic, prevalence of caregivers was 16.50% (*n* = 85).

Mean score of COVID-19-related risk factors was 2.31 ± 0.86 (ranging from 0 to 4). Specifically, 83.50% (*n* = 420) of the sample reported having friends or relatives infected with COVID-19. Moreover, overall prevalence of self-reported COVID-19 symptoms or diagnosis was 45.70% (*n* = 230). Data about occupational status showed that almost 14% (*n* = 69) of the sample were working in a job with direct or high potential exposure to COVID-19. Finally, regarding participants’ psychological status, 15.50% (*n* = 78) reported having received or asked for psychological support during COVID-19 pandemic. According to PCL-5 scores, the mean PTSS severity of the sample was 21.52 ± 12.78, and mean levels of fear of COVID-19 were 18.73 ± 6.08.

### 3.2. Correlations between Study Variables

Correlations between study variables and internal consistency (McDonald’s omega coefficients) are displayed in Table 2. Results showed that fear of COVID-19 was strongly associated with PTSS symptomatology (*r* = 0.59, *p* = < 0.001). A direct association was also found between fear of COVID-19 and informal caregiving (*r* = 0.16, *p* ≤ 001).

Moreover, bivariate correlations indicated that increased levels of fear were associated with female gender (*r* = −0.25, *p* ≤ 0.001) and higher number of COVID-19 risk factors (*r* = 0.13, *p* = 0.003). Conversely, fear of COVID-19 was not associated with age (*r* = 0.08, *p* = 0.070) or educational level (*r* = 0.02, *p* = 0.712).

Higher levels of PTSS symptoms were also associated with caregiving (*r* = 0.18, *p* ≤ 0.001), higher scores in COVID-19 risk factors (*r* = 0.22, *p* ≤ 0.001) and female gender (*r* = −0.12, *p* = 0.009). Unlike fear of COVID-19, results indicated that when age decreased, PTSS symptoms increased (*r* = −0.10, *p* = 0.032).

### 3.3. Moderation Analysis

**Figure 2:**
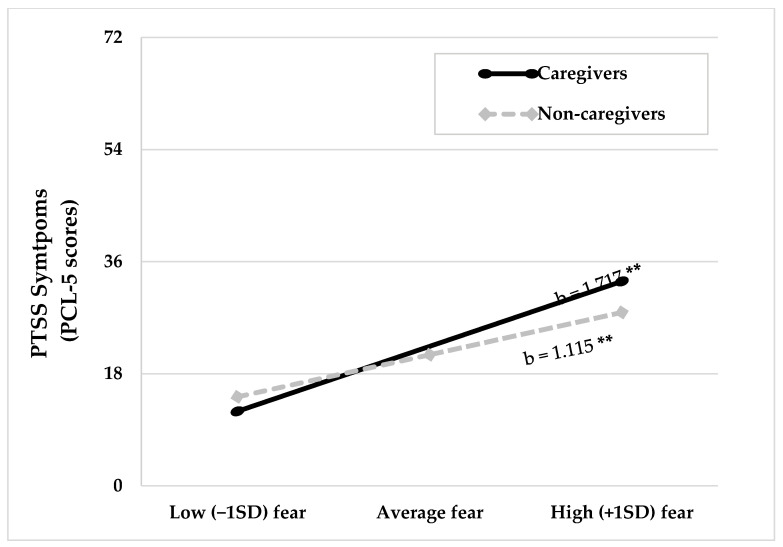
Plots and simple slopes for the significant moderation effect of caregiving. Caregiving moderates the relationship between fear of COVID-19 (displayed on the *x*-axis) and PTSS (displayed on the *y*-axis). Mean of fear of COVID-19 was 18.73 with a standard deviation of 6.08. Simple slope analyses were conducted at three levels of fear: low (one standard deviation below the mean), average (mean value of fear of COVID-19) and high (one standard deviation above the mean). Notes: b = unstandardized coefficients, ** *p* < 0.01.

Table 3 shows the results of the simple moderation analysis. The total model accounted for 39.60% of the variance in PTSS symptomatology. Results indicated that age (b = −0.13, *p* = 0.001) and COVID-19 risk factors index (b = 1.17, *p* = 0.002) significantly predict PTSS symptoms. Although informal caregiving was not significant when predicting PTSS severity (b = 1.34, *p* = 0.289), the interaction between fear of COVID-19 and caregiving was statistically significant in the model (b = 0.60, *p* = 0.001). Simple slopes analyses showed that relationship between fear and PTSS was significant among informal caregivers and non-caregivers. However, fear of COVID-19 had a stronger effect on PTSS symptoms in caregivers (see Figure 2). Simple slope values for the informal caregivers group were b = 1.717 (95% CI = 1.366–2.077, *p* = 0.001), meanwhile the slope values of the non-caregivers group were b = 1.115 (95% CI = 0.949–1.282, *p* = 0.001).

## 4. Discussion

In this cross-sectional study, we hypothesized that informal caregiving during COVID-19 pandemic could moderate the relationship between fear of COVID-19 and PTSS symptoms.

Prevalence rates of informal caregiving (16.5%) found in our sample were similar to the previously reported in Spain [55]. Our first hypothesis assumed that higher levels of fear of COVID-19 and greater PTSS symptoms will be significantly associated with female gender, younger age, lower education levels, informal caregiving, and higher number of COVID-19-related variables. In this regard, we found increased levels of distress in caregivers when compared to non-caregiving population, which is consistent with previous studies [40,43,44,45,46]. Along with prior evidence, our findings also showed that higher levels of fear and PTSS symptomatology were associated with caregiving [17,39,40,41,53].

We also hypothesized that high levels of fear of COVID-19 will be significantly associated with greater PTSS severity. This hypothesis was confirmed since both mental health outcomes (fear and PTSS) were found to be strongly correlated (*r* = 0.59) and mean severity scores were similar to previous studies [64,68,69]. Regarding other variables that could increase psychological distress in our sample, fear and PTSS symptoms were also associated with female gender and higher scores in COVID-19-related risk factors [17,25,27,30,34,35,36,53,70,71]. These risk factors included occupational risk of COVID-19, self-reporting of COVID-19 diagnosis, having relative/friends diagnosed with the disease and being under psychological treatment.

Finally, we also assumed that informal caregiving would moderate the association between fear of COVID-19 and PTSS severity. As hypothesized, the moderation analysis showed that informal caregiving during COVID-19 pandemic affected the strength of the relationship between fear and PTSS symptoms. In this regard, our findings suggest that being a caregiver could increase the fear’s impact on the severity of PTSS. Although we did not find specific studies with which to compare these results, variables associated with caregiving have been found to moderate the development of post-traumatic symptoms after a strong stressor like a natural disaster [72]. Moreover, our regression model showed that a younger age and a greater number of COVID-19 risk factors directly predict PTSS symptoms, which is also consistent with previous findings [73,74].

Several published studies have pointed out the worsening in the care situation during COVID-19 pandemic [43,47,48]. However, the relationship between caregiving and mental health outcomes has been mainly analyzed in a comparative, descriptive manner. These statistical analyses do not allow to conclude about the nature of these associations [75]. For this reason, the findings of this study could be useful to understand when caregiving impacts mental health of the general population during COVID-19 pandemic. In this sense, our results suggest that, even without considering specific caregiving variables, assessing regularly care for a person with a chronic health problem or disability could be useful to detect individuals at higher risk of developing severe PTSS when experiencing fear of COVID-19.

These findings should be seen in the light of several limitations. First, the potential bias associated with the use of self-report measures to assess mental health status. Second, the representativeness of the sample could be improved using a random selection method, since female gender was more prevalent in our study. Nonetheless, higher women’s participation in online surveys regarding COVID-19 pandemic has been also found in previous studies [46,70,76]. Third, findings are cross-sectional which did not allow us to confirm the directionality of fear and PTSS symptoms’ association.

However, to the best of our knowledge, this is the first study that specifically examines the role of informal caregiving during COVID-19 pandemic in the relationship between fear and PTSS. Further studies with larger and more heterogeneous samples are needed to confirm our findings. Moreover, future studies could deepen understanding of identifying caregiver characteristics and variables associated with caregiver burden associated with fear and PTSS development in the context of pandemics.

## 5. Conclusions

Post-traumatic symptoms are the most frequent psychopathological manifestation of fear. This study is a first approximation to determine the role of caregiving in mental health impact of COVID-19 pandemic. We have focused on analyzing when the relationship between fear of COVID-19 and development of PTSS occur, using informal caregiving as the moderator variable. Evidence from this study suggest that regularly care for a person with health problems increases the fear’s impact on mental health status. In this sense, we have found that informal caregivers showed greater PTSS severity in comparison with non-caregivers with same levels of fear of COVID-19. Although further studies are needed, these findings could help to understand in which contexts the relationship between PTSS and fear is stronger. This could be useful to develop targeted treatments and prevention strategies to reduce the risk of developing PTSS in this population.

## Figures and Tables

**Table 1:** Demographic, psychological, and COVID-19 related characteristics of the sample.

Variables	Total (N = 503)	Range
Gender, % (*n*)		
Male	17.50 (88)	
Female	82.50 (415)	
Age, M ± SD	35.54 ± 12.79	18–75
Educational level, % (*n*)		
Elementary/primary	2.40 (12)	
Secondary/technical	40.60 (204)	
University or higher	57.10 (287)	
Informal caregiving, % (*n*)	16.90 (85)	
Fear of COVID-19 (FCVS-19), M ± SD	18.73 ± 6.08	7–35
Post-traumatic stress symptoms (PCL-5), M ± SD	21.52 ± 12.78	0–72
COVID-19 risk factors, M ± SD ^†^	2.31 ± 0.86	0–4
^a^ Potential/direct occupational exposure to COVID-19, % (*n*)	13.70 (69)	
^b^ Friends/relatives infected with COVID-19, % (*n*)	83.50 (420)	
^c^ Reported COVID-19 symptoms/diagnosis, % (*n*)	45.70 (230)	
^d^ Receiving psychiatric/psychological treatment, % (*n*)	15.50 (78)	

^†^ COVID-19 risk factors score is the sum of the positive answers in a, b, c, and d risk factors.

**Table 2:** Bivariate correlations of study variables.

	1	2	3	4	5	6	7
1. Fear of COVID-19	(0.87)						
2. PTSS symptoms	0.587 **	(0.92)					
3. Informal Caregiving (ref. no) ^a^	0.162 **	0.175 **	--				
4. Gender (ref. male) ^b^	−0.247 **	−0.117 **	−0.068	--			
5. Age	0.081	−0.096 *	0.005	0.012	--		
6. Educational level (ref. elementary) ^c^	0.016	0.041	0.005	−0.078	0.094 *	--	
7. COVID-19 risk factors	0.131 **	0.216 **	0.122 **	0.023	−0.165 **	−0.091 *	--

Notes. Correlations were computed using Pearson’s correlation for continuous variables, point-biserial for binary variables and Spearman’s rank for ordinal variables. Reliability coefficients (McDonald’s omega) are displayed in parentheses. ^a^ 0 = non-caregiver, 1 = informal caregiver; ^b^ 0 = female, 1 = male; ^c^ 0 = elementary, 1 = secondary, 2 = university. * *p* < 0.05, ** *p* < 0.01.

**Table 3:** Caregiving as a moderator of the relationship between fear of COVID-19 and PTSS symptoms.

Variables	B (SE)	95% CI	*p*
Fear of COVID-19	**1.115 (0.085)**	**[0.949, 1.282]**	0.001 **
Informal caregiving	1.341 (1.263)	[−1.141, 3.824]	0.289
Fear x Caregiving	**0.602 (0.200)**	**[0.209, 0.995]**	0.003 **
Covariates			
Gender (ref. = male)	0.936 (1.215)	[−1.452, 3.323]	0.442
Age	**−0.127 (0.036)**	**[−0.197, −0.057]**	0.001 **
COVID-19 risk factors	**1.714 (0.538)**	**[0.658, 2.770]**	0.002 **
Constant	**21.459 (1.969)**	**[17.590, 25.328]**	0.001 **

Total variance explained by the model: R^2^ = 0.396 (*p* = 0.001). Notes. B = unstandardized coefficients, SE = standard error, CI = confidence interval. * *p* < 0.05, ** *p* < 0.01. Significant results are displayed in bold.

## Data Availability

The data presented in this study are available on request from the corresponding author.
